# Migrasomes: key players in immune regulation and promising medical applications

**DOI:** 10.3389/fimmu.2025.1592314

**Published:** 2025-05-15

**Authors:** Shuping Li, Yanjie Zhou, Tingting Hu, Mei Li, Yao Luo, Jie Chen

**Affiliations:** Department of Laboratory Medicine/Clinical Laboratory Medicine Research Center, West China Hospital, Sichuan University, Chengdu, Sichuan, China

**Keywords:** migrasomes, immune regulation, biogenesis, intercellular communication, clinical application

## Abstract

Migrasomes are newly discovered extracellular organelles released by migrating cells, such as immune cells, tumor cells, and other special functional cells like podocytes and embryonic cells. They contain a diverse array of constituents, including proteins, lipids, and RNA which can be released to the designated location to activate surrounding cells, thereby facilitating intercellular communication and signal transduction. Since then, our understanding of the mechanism and function of the migrasomes has expanded exponentially, with recent evidence indicating they are involved in various physiological and pathological processes, particularly in immune regulation. Furthermore, methods and techniques for extracting, detecting, and characterizing migrasomes are constantly advancing. Herein, we summarize the current understanding of migrasomes and their key roles in modulating immune responses, as well as the prospective challenges surrounding their clinical application, aiming to provide novel insights into the emerging organelles.

## 1 Introduction

**Figure 1:**
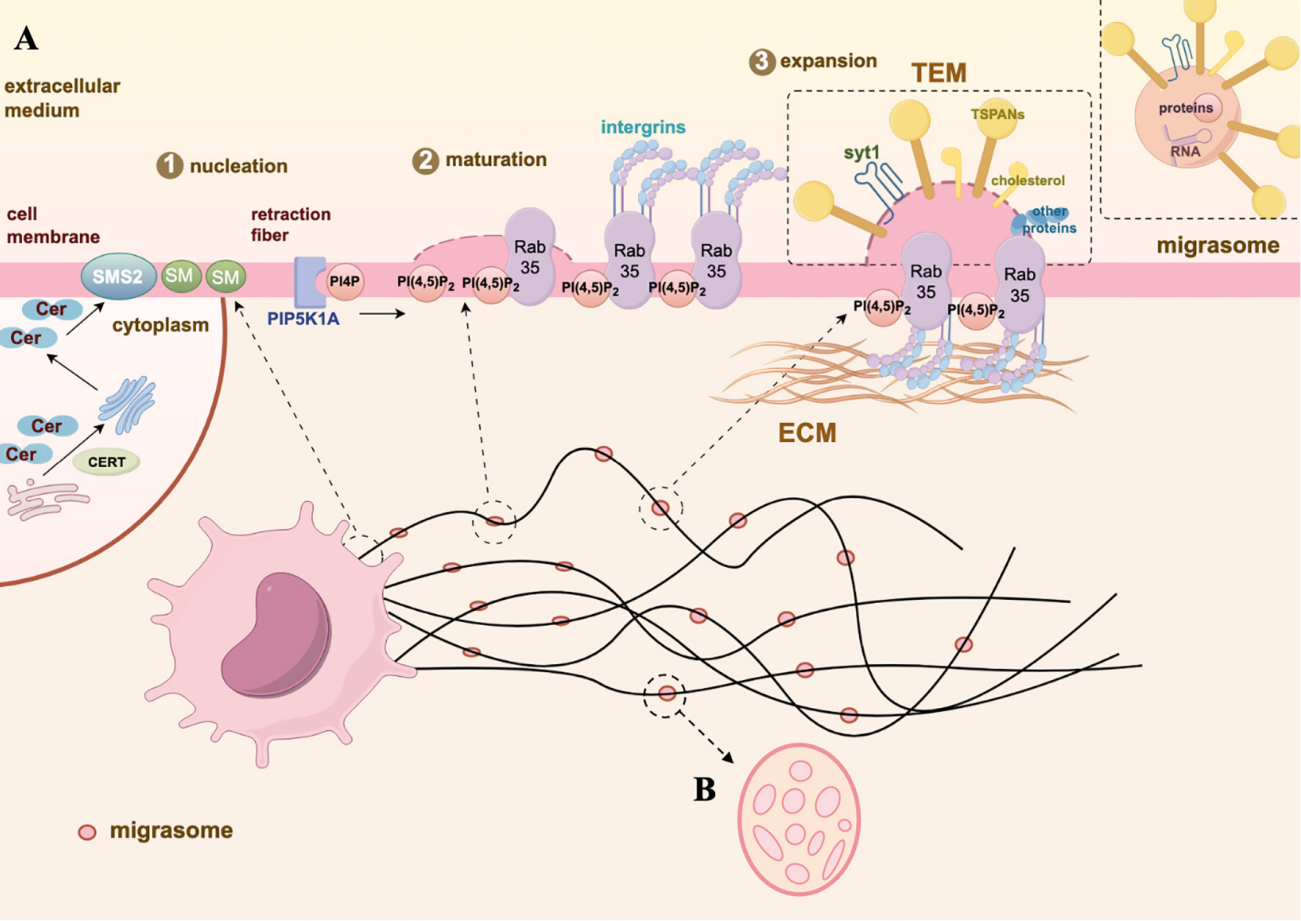
Biogenesis and morphology of migrasome. **(A)** The biogenesis of migrasomes undergoes three stages: nucleation, maturation, and expansion. Nucleation: SMS2, located at the front end of the cell, converts ceramide into SM, gradually moving to the RFs and determining the site of migrasomes formation. Maturation: PIP5K1A converts PI4P into PI(4,5)P_2_. The active PI(4,5)P_2_ promotes the aggregation of integrins by binding with Rab35, simultaneously anchoring the migrasomes to specific ECM proteins. Expansion: TEMs and syt1 accumulate on the membrane of the migrasomes to maintain the membrane’s swollen structure. Subsequently, TSPANs, cholesterol, and integrins further form TEMAs, facilitating the stabilization of the membrane structure and the production of migrasomes. **(B)** The membrane-bound vesicular structure of migrasomes is located on the RFs which in the extracellular space around cells, containing many small vesicles inside, resembling a pomegranate-like structure. The graph is created with https://www.figdraw.com/static/index.html#/.

The term migrasome was first identified by professor Yu Li’s team in 2014 as leaving extracellular organelle related to cell migration, containing cellular contents (1). It has been fully demonstrated that migrasomes are present in many cell types, body fluids and tissues (**Supplementary Table S1**). In addition, migrasomes can be visualized with membrane-bound vesicular structures in the extracellular space around cells by transmission electron microscopy (TEM) and scanning electron microscopy (SEM). The diameter of migrasomes ranges from 500 to 3000 nm. Meanwhile, these vesicles contain numerous smaller vesicles, with diameters of 50–100 nm (1) (**Figure 1B**).

The formation of migrasomes is dependent on cells migration. During the process of cell migration, intracellular substances (such as proteins, lipids, and RNA) can be transported into migrasomes via retraction fibers (RFs). When these migrasomes reach specific locations, the RFs break, and the migrasomes detach from the donor cells. The mature migrasomes then release their carried signaling molecules (such as chemokines, cytokines, growth factors) into the surrounding environment through self-fragmentation or phagocytosis by surrounding cells, thereby impacting the functions of recipient cells and completing intercellular signaling. This phenomenon is termed “migracytosis” (2). Therefore, the migrasomes carry substances from donor cells and have functions closely related to the characteristics of the parent cells, reflecting the traits of the parent cells. Regarding the nature of migrasomes, when they are initially formed, they are connected to the cell via RFs and can perform specific cellular functions. After detaching from RFs, they form a free, independent organelle with information transmission functions similar to extracellular vesicles (EVs). Furthermore, studies have shown that migrasomes encapsulate higher levels of cytokines than the cell body, making them a necessary component and primary source of cytokine in migrating cells. They are considered important carriers of intercellular communication and play roles in various pathological and physiological processes (1). Additionally, migrasomes contain many long-chain mRNAs related to metabolism, intracellular substance transport, and vesicle fusion, which can be laterally transported into recipient cells for translation (3). With the development of biomedical research, migrasomes have been shown to play significant roles in intercellular signal transduction, cell migration, and disease occurrence.

Cell migration refers to the movement of cells after receiving migration signals, such as chemokines (4). In the pathophysiological process, immune cells migration, particularly neutrophils, and monocytes, plays critical roles in proper immune response, wound repair, tissue homeostasis, angiogenesis, inflammatory responses, and tumor progression (5–7). In order to respond effectively to a pathogen during infection, immune cells must first migrate from the blood to the site of infection (8). This process is facilitated by a range of chemical signals, including cytokines and chemokines, released by infected or damaged tissues (4). Many studies have shown that migrasomes with cytokines and chemokines play an essential role in the migration of immune cells, facilitating their development and maintenance to the initiation of primary and humoral immune responses, and aggregating in the abnormal recruitment of immune cells during disease processes. For instance, in inflammatory environment, migrasomes can facilitate the migration of tissue cells and immune cells, serving as a major center for the release of inflammatory factors and regulating the progression of inflammation (9). During cellular physiological processes, migrasomes can also maintain the homeostasis of mitochondrial quality, including the differentiation of and the migration of mature neutrophils from the bone marrow to the circulatory system.

This review provides an in-depth discussion of the biological functions of migrasomes in immune regulation, analyzing their specific roles in intercellular signal transmission, inflammation response regulation, immune cell activation and migration, and tissue repair processes. We also discuss how the signaling molecules carried by migrasomes influence immune responses and intercellular interactions under pathological conditions, thereby providing new perspectives for understanding immune regulatory mechanisms. Through these discussions, we aim to comprehensively reveal the importance of migrasomes in immune responses and their potential clinical applications.

## 2 Discovery and biogenesis of migrasomes

In 1945, by using TEM, Porter KR and colleagues were the first to observe and describe long protruding structures on the cell surface (10). Subsequently, Taylor and Robbins noted long tubular structures extending outward from the migrating cells membrane, which might be related to cell retraction. They named these structures as RFs (11). In 2014, Yu Li’s team observed organelles resembling “pomegranate-like structures” containing numerous vesicles attached to the tips or intersections of RFs by using *in situ* transmission electron microscopy. These structures, measuring 500-3000nm in diameter, were named “pomegranate-like structures (PLS)” (1). Further research found that during cell migration, RFs located at the rear of the cell would break, releasing the attached PLS into the extracellular space. Therefore, PLS is considered a novel type of membrane-bound EVs produced by migrating cells, and it was named “migrasomes” (1).

So how does the biogenesis of this newly discovered vesicular organelles? It is a highly orchestrated process regulated by signaling pathways, rather than simply shedding from cell membrane (**Figure 1A**). Analysis of the lipidomics of migrasomes and cell membranes shows that sphingomyelin (SM) is highly enriched in migrasomes (12). It is known that SM, one of the most abundant lipids in cell membranes, is primarily synthesized from ceramide through the action of sphingomyelin synthases (SMSs) (13). The human sphingomyelinase family includes sphingomyelin synthase 1 (SMS1), sphingomyelin synthase 2 (SMS2), and sphingomyelin synthase-related protein (SMSr) (14). Further analyses reveal that ceramide synthase Cers5, ceramide, ceramide transport protein (CERT), and sphingomyelin synthase 2 (SMS2) are enriched at the sites of migrasome formation (12). The specific process can be described as follows: Cers5 synthesizes ceramide in the endoplasmic reticulum, CERT then transports it to the Golgi apparatus (12, 15). SMS2 attaches to the basal membrane at the leading edge of the cell, converting ceramide delivered to the plasma membrane into SM (12). During cell migration, SMS2 and sphingomyelin move from the cell to the extracellular RFs, predefining the sites for migrasome formation (12, 16). Concurrently, phosphatidylinositol 4-monophosphate 5-kinase 1A (PIP5K1A) also accumulates on RFs, where it phosphorylates phosphatidylinositol 4-monophosphate into the important signal phosphatidylinositol (4,5)-bisphosphate (PI(4,5)P_2_) (17, 18). PI(4,5)P_2_ is recognized present in the plasma membrane and other organelle membranes, as a multi-functional lipid that regulates cell signaling (19). Once PI(4,5)P_2_ reaches the concentration threshold, -binding proteins Rab35, which play a key role in organelle biogenesis, is recruited to the migrasome formation site to regulate migrasome formation. Rab35 then recruit integrin α5 (20), an important adhesion molecule required for migrasomes localization and cell migration (21, 22). A variety of integrins accumulate at the bottom of the migrasomes and provide adhesion for the formation of RFs by binding to specific extracellular matrix (ECM) proteins (23), thus ensuring that the migrasomes produced by cells do not move along with the migrating cells (22). Ultimately, tetraspanin (TSPAN) family proteins, along with integrins and cholesterol, form tetraspanin-enriched macrodomains (TEM), which promote the growth of RFs and the formation of migrasomes vesicular structures (24). Notably, TSPAN4 is one of the most effective proteins in inducing the formation of migrasomes, primarily enriched on the membrane surface (24). Labeling TSPAN4 with green fluorescent protein (GFP) can be used as a characteristic marker to identify migrasomes (24).Cholesterol, an important component in the formation of migrasomes. The enrichment of cholesterol in migrasomes is tenfold larger than in TSPAN4 (24). When using lipoprotein-deficient serum for cell culture, the level of intracellular cholesterol is significantly reduced, which results in a significant decrease in the number of migrasomes (25). Conversely, excessively high levels of cholesterol can also inhibit cell migration and the formation of migrasomes (26). Synaptotagmin-1 (Syt1), as a member of the Ca^2+^-dependent synaptic vesicle membrane protein family, facilitates the exocytosis of synaptic vesicles by binding to Ca^2+^ (27, 28). Ca^2+^ plays a role in the formation of the cytoskeleton and cell adhesion. Recent studies have shown that Syt1 is recruited to the sites of migrasomes formation before the assembly of TEM, generating structurally unstable precursors of the migrasomes (29). The ability of Syt1 to bind Ca^2+^ is crucial for the formation of migrasomes (29). Subsequently, TEM are recruited to the precursors to form stable migrasomes structures (29). These characteristic proteins of migrasomes are crucial for distinguishing them from other EVs, playing a key role in understanding the functions of migrasomes during pathophysiological processes. We sort out the differences between migrasomes and exosomes, which can further understand the biogenesis of migrasomes and their research potential (**Table 1**).

**Table 1:** The distinction of migrasomes and exosomes.

Characteristic	Migrasomes	Exosomes
Size	500-3000nm	30-200nm
Morphology	pomegranate-like structures, large vesicles encapsulating numerous smaller vesicles ranging 50–100 nm (1)	single-membrane vesicles that have a simple, spheroid morphology (30)
Biogenesis	released from the tip of RFs, that at the trailing edge of migrating cells (1)	formed by budding from both the plasma membrane and the endosome membrane (31)
Content	proteins, lipids, RNA, organelles, virus, bacteria and numerous smaller vesicles (1, 22, 25, 32–34)	proteins, lipids, glycoconjugates, RNA, and DNA (30)
Stability	strong interaction with fibronectin and other ECM components through integrin, persisting after disappearance of the RFs (22)	less interaction with ECM, it will not persist stably (30, 31, 35)
Markers	TSPAN4, integrin β1, WGA, CPQ, NDST1, PIGK, EOGT (1, 36, 37)	Tsg101, HSC70, HSP90β, HSP60, CD81, CD9, CD63, CD80, ALIX (38)
Functions	intercellular substance exchange, cell communication, tissue injury repair, maintenance of mitochondrial homeostasis, morphogenesis and embryonic development, inflammatory response, immune regulation, angiogenesis, viral infection, tumor invasion and metastasis (32, 39–47)	intercellular communication, immune response, cell proliferation, homeostasis and maturation, tumor pathogenesis, neurodegenerative pathogenesis and pathogenic infection (30, 48)

ALIX, ALG-2 interacting protein-X; CPQ, carboxypeptidase Q; ECM, extracellular matrix; EOGT, EGF domain specific O-linked N-acetylglucosamine transferase; HSC70, heat shock cognate 70 kda protein; HSP90β, heat shock protein 90 beta; NDST1, N-deacetylase and N-sulfotransferase 1; PIGK, phosphatidylinositol glycan anchor biosynthesis class K; Tsg101, tumor susceptibility gene 101; WGA, wheat germ agglutinin.

However, migrasomes are organelles rather than EVs (49). Firstly, the formation of migrasomes requires the interregulation of multiple molecules and involves three critical stages: nucleation, maturation, and expansion, rather than simply detaching from RFs (50), which membrane fragments are passively lost from the trailing edge of migrating cells. Secondly, migrasomes contain numerous small vesicles and possess their own membrane structure and characteristic morphology, similar to other organelles. Additionally, migrasomes can be produced by various cell types and widely present in bodily fluids and tissues (51). Most importantly, migrasomes encapsulate higher levels of cytokines than the cells, which are essential components and primary sources of cytokine secretion from migrating cells, playing an important roles in various pathophysiological processes (9).

## 3 Molecular composition of migrasomes

Migrasomes, as an extracellular organelle secreted by cells, not only contain lipids and proteins essential for the formation, but also include numerous small vesicles and bioactive substances internally. Mass spectrometry analysis of the purified migrasomes revealed that the proteins within these structures can be categorized by localization into membrane proteins, nucleus proteins, cytosol proteins, extracellular proteins, lumen proteins and nucleus proteins (1). Based on functional classification, they may include contractile proteins, DNA interaction proteins, complement system proteins, signaling factors, enzymes, cytoskeleton proteins, receptors, chaperones proteins, vesicle traffic proteins, cell adhesion proteins, RNA binding proteins and Others (1) (**Table 2**).

**Table 2:** Composition of migrasomes.

Subcellular localization	Function	Name	References
membrane	cytoskeleton	tetraspanins, cholesterol, SM, PI (4,5)P_2_, actin and tubulin	(1, 12, 17, 22, 24, 52, 53)
	adhesion	integrins	(22)
	enzymes	SMS2, Rab35	(12, 16)
intracapsular	chemokines	CXCL12, CXCL10, CXCL8, CXCL5, CCL2, CCL1, CX3CL1, CCL27, CCL21	(33, 39, 43)
	growth factors	M-CSF, VEGFA, Wnt5b, Wnt8a, Wnt11, PDGFD, BMP2, BMP7a	(40, 44)
	morphogens	MYDGF, BMP1, LEFTY1	(39, 40)
	cytokines	TGF-β, IL-1β, IL-10, TNF-α and IL-6	(9, 33)
	coagulation factors	prothrombin, factor XIII B, factor X, factor VIII, factor XI, factor XII and Von Willebrand factor	(54)
	enzymes	Rab35, Rab7, Rab32,Rab2b	(17, 43)
	RNA	RNA and mRNA	(3, 55)
	virus	CHIKV-nsP1, HSV-2, vaccinia virus	(34, 47, 56)
	bacteria	dermcidin	(57)
	organelle	mitochondria	(32, 58)
	others	neuronal fragments	(59)

BMP, bone morphogenetic protein; HSV-2, herpes simplex virus type 2; LEFTY1, left-right determination factor 1; M-CSF, macrophage colony-stimulating factor; MYDGF, myeloid-derived growth factor; PDGFD, platelet-derived growth factor D; PI(4,5)P_2_, phosphatidylinositol (4,5)-bisphosphate; SM, sphingomyelin; SMS2, sphingomyelin synthase 2.

Regarding the composition of the migrasome membrane, as previously mentioned, the microdomains rich in TEM formed by TSPAN4, integrins, and cholesterol are important scaffolds for migrasome formation (24). TEM further enriches on RFs to become macrodomains rich in tetraspanin proteins (TEMA), which can promote vesicle expansion and migrasome formation. The TSPAN family consists of structure-related molecular proteins with four transmembrane domains, distributed in specific areas of the membrane (60). Different ligands interact with TSPANs to participate in cell adhesion, migration, immune responses, and intercellular signaling (61, 62). TSPAN4 is highly enriched on migrasomes and is considered a biomarker for their identification (52). Integrins are heterodimers composed of α and β chains, with different integrins binding to various ECM proteins, mediating adhesion between extracellular matrix proteins and cells (21, 23, 63). Mass spectrometry analysis shows that integrin α5β1 is highly expressed on migrasomes while showing low expression levels on RFs, making it a superior biomarker for migrasomes compared to TSPAN4 (22). Cholesterol, as a crucial lipid component of TEM, is essential for the formation and maintenance of the vesicular structure of migrasomes (1, 64). The significance of other lipid components on the membrane, such as ceramides, SM, and PI (4,5)P_2_ for the biological function of migrasomes has yet to be elucidated. However, ceramides, sphingolipids, and sphingoid bases are involved in various cellular pathways, including apoptosis, cell differentiation, and immune responses (65, 66). It can be speculated that these lipids may act as signaling molecules that influence the function of migrasomes.

Recent studies indicate that during cell migration, vesicles containing a high concentration of signaling molecules, including chemokines, cytokines, and growth factors, polarize to the rear of the cell and are actively transported into migrasomes. This transport process is driven by the actin-dependent motor protein myosin-5a (9, 67). These signaling molecule-containing vesicles fuse with the migrasome membrane through a SNARE-mediated mechanism, releasing their contents into the intercellular space in a manner similar to synaptic vesicles (9, 68, 69). The concentrations of cytokines within migrasomes and their secretion rates are higher than those in the cell body (9). These signaling molecules packaged into migrasomes determine their functions, making migrasomes the primary sites for cytokine secretion in migrating cells (9). For example, during the formation of the embryonic gut in zebrafish, mesoderm and endoderm cells produce migrasomes rich in signaling molecules, including morphogens, growth factors, and cytokines (39). These migrasomes accumulate under the embryonic shield, releasing CXCL12 to attract dorsal progenitor cells (DFCs) toward them (39, 50, 70). The aggregation of DFCs plays a crucial role in the formation of Kupffer’s Vesicle (KV), an important organ in zebrafish embryonic development (71). Importantly, these migrasomes can target and deliver their internal signaling molecules to spatially specific locations to regulate the left-right symmetry of KV development (39, 50, 70). Additionally, in the capillaries of the chorioallantoic membrane (CAM) of chicken embryos, migrating monocytes produce migrasomes rich in VEGFA and CXCL12 along their path ^1^ . These migrasomes create a favorable microenvironment for angiogenesis in the CAM. In summary, migrasomes can act as carriers of signaling molecules and control their spatial distribution, becoming a source of signaling molecules that promote intercellular communication (9). Furthermore, migrasomes also contain damaged mitochondria (32). Under mild mitochondrial stress, damaged mitochondria in the cell can be selectively transported into migrasomes and subsequently released extracellularly, maintaining mitochondrial homeostasis (32, 58). Besides proteins and organelles, migrasomes also contain RNA, including small RNAs and mRNAs (3). These enriched mRNAs in migrasomes are associated with various cellular processes related to metabolism, cell transport, cell adhesion, vesicle fusion, and subcellular and membrane structure assembly (3). Analysis of Pten mRNA contained in migrasomes shows that co-culturing migrasomes expressing PTEN mRNA with PTEN knockout tumor cells effectively restores PTEN expression in tumor cells, subsequently inhibiting pAKT signaling and cell proliferation (3, 72, 73). In contrast, migrasomes lacking PTEN mRNA did not produce this effect. This indicates that mRNA within migrasomes can be laterally transferred to recipient cells and translated into functional proteins, thereby altering the function of the recipient cells. In conclusion, the components within migrasomes can be summarized into three parts: signaling molecules that mediate intercellular communication, such as chemokines and cytokines; slightly damaged mitochondria to ensure mitochondrial homeostasis within the cell; and mRNAs that can be laterally transported to recipient cells and translated. Therefore, the mRNA and proteins enriched in migrasomes are critical mechanisms by which migrasomes exert their physiological functions, and studying this mechanism’s biological functions in organisms will remain a focus for the future.

## 4 The roles of migrasomes in immune regulation

### 4.1 Functions of migrasomes in regulating immune activation and intercellular communication

Migrating immune cells can produce migrasomes that carry signaling molecules, facilitating signal transduction between cells and cells, cells and the ECM, thereby regulating the migration and activation of immune cells (74).

Bacterial infections can induce the activation of immune cells and the release of cytokines, which play a crucial role in inflammation, cell proliferation, differentiation, and the aggregation of immune cells. In the early immune response, dendritic cells (DCs), as the primary antigen-presenting cells, can capture exogenous antigens through various mechanisms, including phagocytosis, endocytosis, and membrane protrusion (75–78). Using polyacrylic acid-coated semiconductor quantum dots as fluorescent artificial antigens (FAAs) to study the membrane fiber network structure shows that DCs can capture FAAs by producing RFs and migrasomes. These migrasomes can be further swallowed by other DCs, mediating intercellular interactions (79). Additionally, RFs can influence the motility of DCs, with the complex and extensive membrane network surrounding the cells increasing the area available for antigen capture and facilitating long-distance intercellular communication (79, 80). Post-stroke pneumonia, which can occur after bacterial infection in patients with acute ischemic stroke (AIS), is one of the major causes of death from AIS (57, 81, 82). Bone marrow mesenchymal stem cells (BM-MSCs) enhance the expression of RUBCN (RUN domain and cysteine-rich domain-containing Beclin 1-interacting protein) in bone marrow-derived (BMDM), promoting its binding to BECN1 and facilitating the assembly of the PI3K complex. This process subsequently activates the essential component of macrophage LC3-associated phagocytosis (LAP), NADPH oxidase (NOX) (83). By enhancing macrophage LAP activity, BM-MSCs improve the bacterial clearance capacity and increase the survival rate of stroke patients following Escherichia coli (E. coli) infected (84). However, BM-MSCs only remain in the lung for approximately 24 hours. The antibacterial effects of injected BM-MSCs remain evident for at least three days, which is associated with the migrasomes they produced (57). These migrasomes can persist in the lungs of AIS patients for an extended period and enhance bacterial clearance by upregulating the expression of RUBCN in BMDM (57, 84–87). Furthermore, the migrasomes produced by BM-MSCs are enriched with high concentrations of the antimicrobial peptide dermcidin (DCD), which significantly reduces lung bacterial load and enhances macrophage LAP following E. coli infection (57). Although DCD can directly disrupt bacterial cell membranes to exert a bactericidal effect (86), BM-MSC-derived migrasomes containing DCD exhibit a higher bacterial killing rate, indicating their crucial role in the immune response to bacterial infections. Intraperitoneal injection of E. coli or lipopolysaccharide (LPS) significantly increases the number of migrasomes produced by neutrophils in mice, with the quantity of migrasomes induced by bacterial infection being significantly higher than that induced by LPS (54). The clostridial toxins TcdA and TcdB are potent exotoxins produced by Clostridia (88), which can induce cells to produce chemokines and cytokines, leading to early inflammatory response (89–92). Injection of TcdB3 into the mice peritoneum induces a large production of migrasomes by hepatic sinusoidal endothelial cells and kupffer cells, releasing cytokines (IL-6, CXCL12, and TNF-α) that promote inflammation and drive more neutrophils to accumulate in the hepatic sinusoids, mediating the early immune response (33). Compared to wild-type mice, mice with deletion of TSPAN9, a key protein for the formation of migrasomes, have a longer survival time and a higher survival rate after TcdB3 treatment, with reduced migrasomes production, inhibited plasma monocytes infiltration, and lighter early inflammatory reaction (33). Additionally, following LPS stimulation, monocytes in the peripheral blood can also produce migrasomes enriched with TNF-α and IL-6, which rapidly accumulate at the site of inflammation, becoming a major source of cytokine secretion (93). The rate of migrasomes formation directly influences the amount of cytokine secretion (93). However, the expression level of total cytokines in the blood of mice with TSPAN9 knocked out is significantly reduced (93). Therefore, migrasomes produced by cells not only activate and recruit immune cells but also rapidly transport high concentrations of cytokines to sites of inflammation, serving as a continuous source of inflammatory cytokines and playing a critical role in immune and inflammatory responses. This suggests that migrasomes seem to act as amplifiers for mother cells, regulating various microenvironmental states, and their functions mainly depend on the cytokines encapsulated within the mother cells.

After immune activation, the cytokines produced by immune cells can also regulate the proliferation and migration of vascular endothelial cells to promote angiogenesis. The main protein of migrasomes, TSPANs, is widely expressed in the cardiovascular system and is involved in maintaining vascular homeostasis (94–97). Under physiological conditions, monocytes on the CAM of chicken embryos can produce migrasomes rich in pro-angiogenic factors VEGFA and CXCL12 during migration (40). These migrasomes deliver pro-angiogenic factors to areas of capillary formation, creating a favorable microenvironment for angiogenesis and playing an important role in this process. Additionally, these migrasomes can further recruit monocytes along the cell migration trajectory through CXCL12-mediated chemotaxis, creating a positive feedback loop that rapidly enhances capillary formation both in the CAM and *in vitro* (40). Knockout of TSPAN4 reduces the formation of migrasomes and inhibits monocyte aggregation and capillary formation (40). *In vivo* imaging shows that neutrophils produce RFs and migrasomes while migrating along the hepatic blood vessels in mice (98). These migrasomes can adhere to blood vessels for a long time and be taken up by nearby neutrophils, or they can be separated from blood vessels and enter the circulatory system to participate in the signaling between immune cells (98). Once in the circulatory system, these migrasomes, with their unique cholesterol ester components, can adsorb high concentrations of coagulation factors and adhesion molecules from plasma, exhibiting platelet-like coagulation functions (54). In cases of injury, the migrasomes produced by circulating neutrophils also possess a high-affinity active form of integrin β1, which allows for rapid adhesion and accumulation at the site of injury by binding to collagen, thereby initiating the coagulation cascade (54). These migrasomes have platelet-like functions, activating platelets and the clotting system with significantly superior efficacy to thrombin, thereby promoting the formation of blood clots (54). Exogenous supplementation of these migrasomes can restore coagulation dysfunction caused by neutrophil deficiency or TSPAN9 knockout, enhancing platelet thrombus formation at the site of injury and reducing blood loss (54). Therefore, migrasomes may serve as key regulatory factors in angiogenesis and vascular repair, maintaining vascular homeostasis and influencing disease progression.

Moreover, appropriate immune activation is crucial for effective tissue repair (99–102). Microglia are the primary immune cells in the central nervous system, performing various functions, such as phagocytosing cellular debris and invading pathogens, presenting antigens, and releasing cytokines (103–106). In mice model of mild closed head injury, neutrophils rapidly migrate from the peripheral circulation into the brain parenchyma, adhering to microglia and producing migrasomes (41). These migrasomes can not only act as a traction to make microglia aggregate towards the injury site, but also serve as signaling carriers that can be engulfed by microglia, mediating intercellular interactions (41). This is the first time that the function of migrasomes has been discovered in the mammalian brain, indicating that neutrophils from the peripheral immune system can directly transmit signals to specific immune cells in the central nervous system, such as and monocytes, thereby enhancing neuroimmune functions and promoting tissue repair (41, 99, 107, 108). The formation of migrasomes is related to the binding strength of integrins to the ECM, and conventional studies typically culture migrasomes on fibronectin-coated substrates (109). Research has shown that nanotopography can influence the adsorption of ECM proteins, thereby affecting their binding to integrins and cell behavior, serving as substrates for migrasomes cultivation (42, 109–111). Titania nanotube arrays as substrates can induce M2 polarization of macrophages and the production of migrasomes (42). These migrasomes derived from M2 macrophages express PI3K and AKT at the same high phosphorylation levels as the mother cells. BM-MSCs activate the PI3K-AKT signaling pathway and promote osteogenic differentiation by phagocytosing these migrasomes (42, 112–115). Injection of these migrasomes *in vivo* can promote new bone formation and increase the density and thickness of mature bone, indicating that migrasomes produced by M2 play a role in facilitating bone tissue regeneration (42). This suggests that migrasomes produced by immune cells can assist damaged tissues in re-establishing their structure and function.

However, excessive immune activation may lead to heightened complement activation, exacerbating cytotoxic effects that cause tissue damage. In patients with cerebral amyloid angiopathy (CAA), amyloid protein beta 1-40 (Aβ40) typically accumulates in small blood vessels and capillaries (116–118). However, can phagocytose and clear the deposited Aβ40 (119–121). Research has found when are stimulated by high concentration of Aβ40 in the blood vessels, they can not only phagocytose Aβ40, but also release migrasomes containing complement activation-related molecules, CD5 antigen like (CD5L) (122, 123). These migrasomes migrate from the peripheral blood to the brain parenchyma and the eyes, adhering to endothelial cells and raising local CD5L concentrations to damage levels, attracting complement C5b-9 to co-localize with them on cerebral vessels, thereby inflicting damage to the blood-brain barrier through complement-dependent cytotoxicity (CDC) (123). Plasma protein NeFL expression levels are positively correlated with cognitive dysfunction, and injection of migrasomes produced by Aβ40-stimulated Raw264.7 cells into mice results in elevated levels of plasma NeFL and C5b-9, further indicating that macrophage-derived migrasomes contribute to brain damage via CDC (123). Plasma C5b-9 levels may serve as potential biomarkers for diagnosing CAA (123). Using complement inhibitors like PMX-53 can suppress plasma C5b-9 and Aβ40 expression in CAA mice, alleviating blood-brain barrier damage and CAA progression, offering new therapeutic avenues for CAA treatment (123).

**Figure 2:**
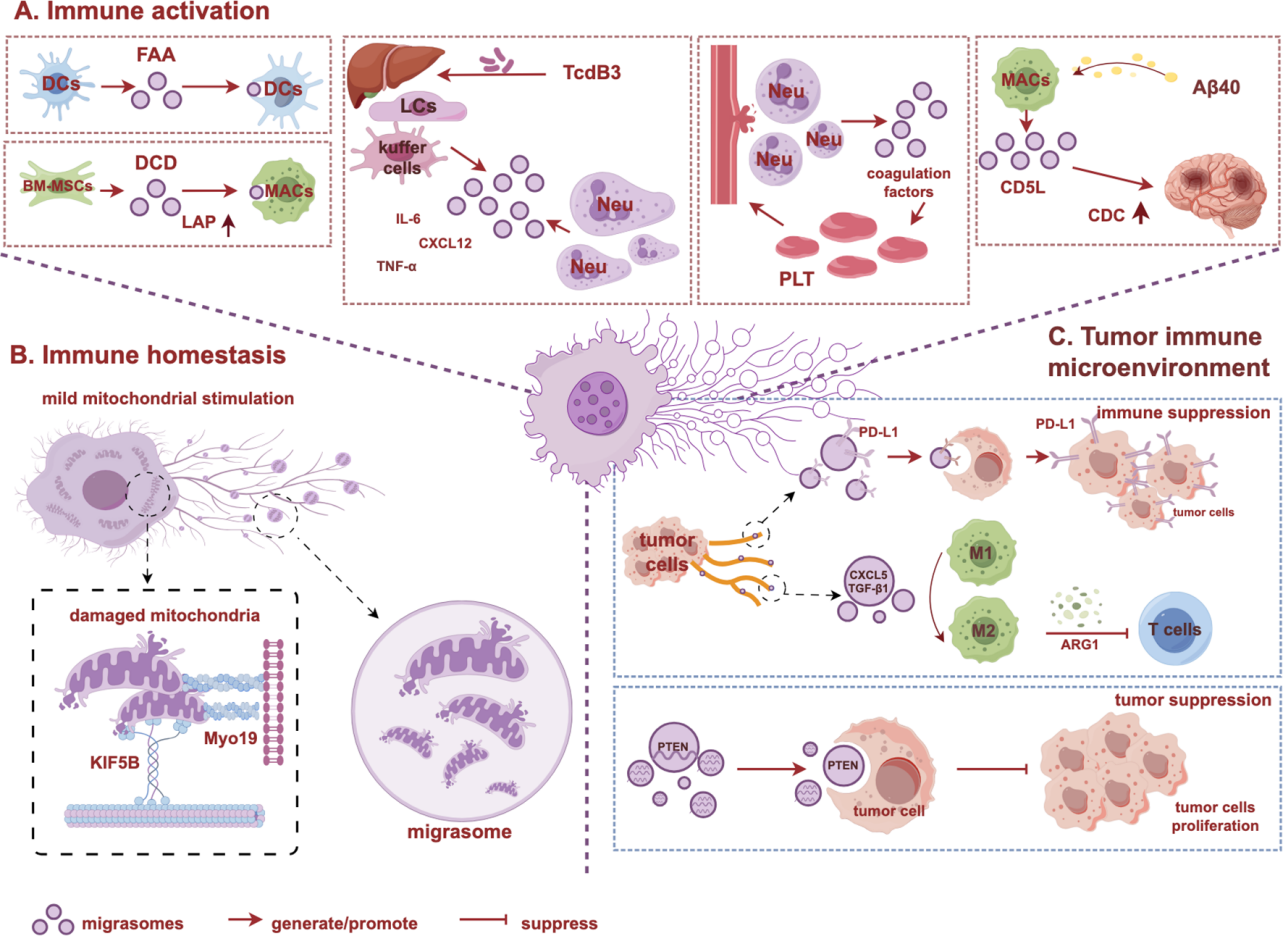
Biological functions of migrasomes in immune regulation. **(A)** Immune activation: migrasomes can serve as carriers to activate immune cells, mediating intercellular information communication and regulating immune responses. **(B)** Immune homeostasis: migrasomes remove damaged mitochondria from the cell, maintaining immune cells mitochondrial homeostasis. **(C)** Tumor immune microenvironment: migrasomes play dual roles in regulating tumor microenvironment. The graph is created with https://www.figdraw.com/static/index.html#/. BM-MSCs, bone marrow mesenchymal stem cells; CDC, complement-dependent cytotoxicity; CD5L, CD5 antigen-like; LAP, LC3-associated phagocytosis; LCs, liver cells; Neu, neutrophil; MACs, macrophage; Myo19, Myosin 19; KIF5B, kinesin family member 5B.

In summary, migrasomes may serve as facilitators for mother cells in mediating immune activation, and their function is highly dependent on the type of mother cells, the contents they carry, and environmental signals. Migrasomes derived from BM-MSCs primarily play roles in antibacterial activity and tissue repair, while migrasomes sourced from monocytes/or neutrophils are more inclined to release pro-inflammatory factors during inflammation. Under physiological conditions, migrasomes promote angiogenesis, maintain vascular homeostasis, and regulate coagulation processes by transmitting contents. However, in pathological conditions, the functions of migrasomes are reshaped by abnormal microenvironments into a “double-edged sword,” which can both enhance immune responses to suppress bacterial infections and, through excessive activation, lead to tissue damage. In summary, the secretory mechanisms mediated by migrasomes from different cell types are universal across various cells. By integrating biological and spatial signals, they transmit combined signals and mediate intercellular communication, influencing and altering the behavior and status of recipient cells, thus promoting interactions between cells (**Figure 2A**). The occurrence and progression of diseases involve multiple pathological processes, closely linked to interactions between different cell types and immune cells, making the role of migrasomes indispensable.

### 4.2 The roles of migrasomes in maintaining cell viability and immune homeostasis

Activated immune cells exhibit higher migration and respiration rates, require increased energy consumption, produce more reactive oxygen species, and respond to higher mitochondrial stress. Migrasomes help maintain mitochondrial homeostasis in these immune cells (**Figure 2B**). When mouse fibroblasts were treated with the 2 μM oxidative phosphate uncoupling agent carbonyl cyanide 3 chlorophenylhydrazone (CCCP) to induce mild mitochondrial stress, the damaged mitochondria binded to the kinesin family member 5B (KIF5B) and moved to the plasma membrane, where they were split into fragments by dynein associated protein 1 and adhered to the plasma membrane area via Myosin 19 (Myo19) (32, 124). During cell migration, these fragments entered migrasomes, forming a type of migrasomes termed “mitosome,” which released mitochondria through a process known as mitocytosis. This process regulates mitochondrial balance during mild mitochondrial stress and stabilizes mitochondrial membrane potential (MMP) (32). However, when cells were treated with 10 μM of CCCP, intense mitochondrial autophagy signals are activated. At this time, the cells do not migrate, and the phenomenon of mitochondrial exocytosis does not occur (32). Therefore, mitochondrial autophagy and mitocytosis work together to maintain mitochondrial quality under different stress conditions (32, 58, 125, 126). Overexpression of TSPAN4/9 improves the low potential state of MMP by increasing migrasomes formation, whereas TSPAN9 knockout mice results in reduced migrasomes production and decreased mitochondrial MMP levels, leading to impaired neutrophil viability, indicating that the survival and migration of circulating neutrophils require mitocytosis to be sustained (32, 58). Similarly, the stability of mitochondrial quality in bone marrow-derived during differentiation also relies on mitocytosis (32). As a newly discovered quality control mechanism for mitochondria under low-stress conditions, mitocytosis regulates mitochondrial quality by removing damaged mitochondria, maintaining mitochondrial homeostasis in circulating immune cells during differentiation and migration, and preserving cell viability (32, 58, 127).

### 4.3 The dual roles of migrasomes in regulating tumor microenvironment

Tumor cells possess enhanced migration and invasion capabilities (5, 128, 129) and are considered a primary source of migrasomes in tumor tissues. Analysis of gene expression data from various cancers reveals that migrasome-related genes, particularly the migrasomes marker protein PIGK, show higher expression levels in multiple tumor tissues, with an increase corresponding to tumor progression (130). Moreover, overexpression of migrasomes genes indicates poorer prognosis and lower survival rates, which is associated with the ability of migrasome-producing tumor cells to foster an immunosuppressive tumor microenvironment (TME) (130). The differential membrane tension between the front and rear of cells is a critical factor for maintaining continuous cell migration (131). In migrating tumor cells, programmed death ligand 1 (PD-L1) activates RhoA by localizing integrin β4 at the rear of the cell and binding to the actin at the rear, promoting RhoA-mediated contractility and maintaining lower membrane tension at the rear, thereby regulating cell polarity and sustaining the continuous migration of tumor cells (132, 133). The high expression of PD-L1 at the rear of breast cancer cells (MDA-MB-231) is a key factor promoting persistent cell migration, and this high level of PD-L1, along with integrin β4, primarily concentrates in the RFs and migrasomes formed in the cell tail (133). Neighboring cells can phagocytose these PD-L1-containing migrasomes, increasing their own PD-L1 expression and facilitating immune evasion by tumor cells (133). The non-phagocytic migrasomes release internal cytokines through classical signaling pathways, promoting the migration and aggregation of tumor and stromal cells within the TME, thereby facilitating tumor progression (133). TIMER database reveals that in most tumors, migrasomes are positively correlated with M2 macrophage expression (130). Migrasomes produced by pancreatic cancer cells can significantly increase M2 in mice while reducing CD4+ T lymphocytes, creating an immunosuppressive TME (43). This occurs because migrasomes produced by pancreatic cancer cells are rich in CXCL5 and TGF-β1, promoting macrophage migration into the TME and further phagocytosis of migrasomes, inducing their conversion to the M2 phenotype (134), which ultimately leads to a significant increase in M2 macrophage expression levels in mice (43). These highly express pro-tumor molecules, promoting the proliferation, migration, and invasion of tumor cells (130, 134–136). Furthermore, secrete immunosuppressive molecules, such as ARG1, that may inhibit the activation and proliferation of T cells, thereby promoting the formation of immunosuppressive TME and further promoting the progression of pancreatic cancer (130, 137). However, the mechanism remains to be further studied. CD151, a member of the TSPAN family, is expressed at higher levels in highly metastatic liver cancer cell lines (138–141), leading to increased migrasomes production (44). These migrasomes have a signal localization effect, which can attract neighboring liver cancer cells towards them and promote phagocytosis, so that the cells that phagocytose the migrasomes can acquire the invasion ability similar to highly metastatic liver cancer cells, facilitating the invasion and metastasis of hepatocellular carcinoma (HCC) (44). In addition, angiogenesis plays a crucial role in promoting early cancer metastasis. Just as monocytes produce migrasomes rich in VEGFA and CXCL12 to promote angiogenesis under physiological conditions, migrasomes from highly metastatic liver cancer cells also have high concentrations of VEGFA, further promoting angiogenesis and distant metastasis in mice with liver cancer (40, 44). This indicates that the role of migrasomes is related to the microenvironment. However, the regulatory effect of migrasomes on the TME is not straightforward. PTEN, recognized as a tumor suppressor (72, 73, 142), is one of the most abundant mRNAs in migrasomes (3). Migrasomes with high PTEN expression can inhibit pAKT activity in human breast cancer cells, thus suppressing tumor cell proliferation (3). This paradoxical effect may be because these migrasomes are produced by fibroblasts cultured *in vitro*, rather than originating from tumor cells within the TME. This suggests that the effects of migrasomes on tumor cells are closely related to the proteins and mRNAs enclosed within them, which can alter the functional responses of recipient tumor cells through various pathways. In summary, migrasomes have a regulatory effect on cancer cells within TME (**Figure 2C**). Exploring the sources and contents of migrasomes during disease progression is crucial for understanding their role in disease progression and tumor development.

In summary, migrasomes play a crucial role in immune activation, immune homeostasis, and the tumor immune microenvironment (**Figure 2**). These functions result from the dynamic interactions among the cellular source of migrasomes, their contents, and the microenvironment in which they exist. For example, migrasomes carrying the same molecule, such as VEGFA, promote angiogenesis and embryonic development under physiological conditions but lead to pathological angiogenesis and increased invasiveness in tumors (40, 44). Similarly, migrasomes derived from macrophages contain DCD under bacterial infection, exerting antibacterial effects, while in the presence of CAA, they can exhibit complement toxicity (57, 123). Therefore, the functions of migrasomes are not inherently “good” or “bad” but are determined by their context of generation. The cellular source influences the functional tendency of migrasomes. Normal cells, such as BM-MSCs and monocytes, produce migrasomes that focus on clearing pathogens, repairing tissues, and promoting embryonic development and angiogenesis (40, 42, 57). Pathological cells, such as cancer cells and activated, produce migrasomes that carry pro-cancer factors, complement activation molecules, or inflammatory mediators, driving aberrant angiogenesis, inflammatory storms, or complement-mediated tissue damage (43, 44, 123). Microenvironmental dictate the functional direction of migrasomes. In a stable microenvironment, the physicochemical properties of the ECM regulate the rate of migrasomes formation through integrins, promoting bone tissue regeneration (42). In pathological microenvironments, chronic inflammatory factors (such as IL-6 and TNF-α), or pathological protein deposition (such as Aβ40) alter the metabolism and migration behavior of mother cells, leading to the abnormal enrichment of pro-inflammatory molecules in migrasomes, resulting in “dysregulated” functions (33, 93, 123). Thus, migrasomes amplify local signals briefly to restore homeostasis under physiological conditions, but in disease states, persistent pathological signals may convert the contents of migrasomes to destructive ones, lacking negative feedback mechanisms to terminate their action, leading to adverse outcomes. In conclusion, the core role of migrasomes is to serve as a “response-amplifying” tool for mother cells in response to external signals.

## 5 The clinical application of migrasomes detection methods

Migrasomes not only play a role in immune regulation, but numerous studies have also found their association with specific disease states (**Supplementary Table S1**). For instance, high-salt diet can promote the formation of migrasomes in the brains of mice, further exacerbating ischemic brain injury (59). In proliferative vitreoretinopathy, the migrasomes produced by retinal pigment epithelial cells are linked to disease progression (143). Podocytes can release “damage-associated” migrasomes, which may serve as a novel early diagnostic marker for urinary podocyte injury (144, 145). Migrasomes can also interact with immune cells and signaling molecules in the tumor microenvironment, influencing tumor development (44). They are involved in other physiological and pathological processes, including embryogenesis (39), angiogenesis (40), tissue repair and regeneration (45), inflammatory responses (33), virus infections (34), tumor invasion and metastasis (43). In summary, migrasomes play a crucial role in intercellular communication and serve as a major site for the secretion of cytokines by migrating cells (9). The secretions of migrasomes can reflect key information from their parent cells. Studying the development of migrasomes and their roles in diseases holds the promise of providing new biomarkers for ‘cellular biopsy’. Additionally, migrasomes exhibit low immunogenicity and possess certain biocompatibility. Their bilayer lipid structure can protect the internal cargo from degradation, making them promising carriers for therapeutic drugs and potential players in clinical applications.

However, current research findings remain unclear regarding the specific mechanisms by how migrasomes participate in disease development, primarily due to the inadequacies in existing methods for studying migrasomes. It is critical to understand their functions in disease states and evaluate the application of their detection techniques. Currently, the research methods for migrasomes mainly include three parts: extraction and purification, imaging observation, and in-depth characterization (36). These methods not only promote our in-depth understanding of the biological characteristics of migrasomes, but also provide new possibilities for the early diagnosis and treatment of diseases.

### 5.1 Extraction and purification of migrasomes

Similar to the isolation of EVs, migrasomes can be extracted from body fluids and cell culture-medium using a combination of ultracentrifugation and density gradient centrifugation methods (2, 146–148). Cell debris and large cell fragments should be removed using trypsin and low-speed centrifugation prior to the isolation of migrasomes to reduce contamination of crude samples. Subsequently, density gradient centrifugation is employed to enrich the migrasomes (2). Throughout the isolation process, maintaining cells integrity as much as possible is critical to avoid the release of organelles from ruptured cells, which could affect the purity of the migrasomes (2). Specific isolation methods can be found on Professor Yu Li’s research group website (https://liyu-lab-tsinghua.github.io/protocols/). Due to the relatively low abundance of migrasomes in blood, along with the presence of numerous cells, proteins, and inorganic ions, these components may co-precipitate with migrasomes during centrifugation, thereby impacting the purity of the migrasomes and the accuracy of subsequent detection results. Consequently, traditional isolation methods have certain limitations when applied to clinical blood samples. Moreover, when observing the migrasomes produced by, Ma found a type of migrasome-derived nanoparticles (MDNPs), which have a morphology similar to EVs. These particles can be released from migrasomes through mechanisms such as migrasomes rupture, RFs breakage, or membrane-like budding, subsequently affecting the purification of migrasomes (37). However, the miRNA expression profile of MDNPs differs from that of migrasomes, and their average diameter is approximately 165.3 ± 3.1 nm, significantly smaller than the 500–3000 nm size range of migrasomes (37). Using a 0.45 μm filter to filter the supernatant can effectively separate migrasomes, further purify and reduce the pollution of MDNPs (37). Nevertheless, these separation methods have large sample size requirements, complex operation, time consuming, high cost, low recovery rate, and lack of standardization, which limits their widespread use in practical application. The key to efficiently enriching migrasomes lies in the high specificity recognition of targets. Currently, the main molecules used for target recognition are antibodies and nucleic acid aptamers. Nucleic acid aptamers, also known as chemical antibodies, are short oligonucleotide molecules that can form unique secondary structures and specifically bind to various targets, including cells and pathogens (149–151). In recent years, due to their comparable specificity and affinity to antibodies, along with advantages such as lower cost, higher stability, lower immunogenicity, and ease of chemical modification, aptamers have been widely applied in various biomedical fields (150–152). Therefore, the method of aptamer modification is expected to be used to extract migrasomes.

### 5.2 Characterization and analysis of migrasomes

#### 5.2.1 Visualization imaging

For the extracted specimens, transmission electron microscopy, fluorescence microscopy and scanning electron microscopy can be used to observe the ultrastructure of migrasomes (153). By utilizing GFP or mCherry to label migrasome-specific proteins TSPAN4 and integrins, it is possible to observe the migrasomes in live cells in real-time fluorescence confocal microscopy (153). However, this method is time-consuming, and the overexpression of labeled proteins may affect the natural biosynthesis of migrasomes. To address these issues, Yu Li’s group discovered that wheat germ agglutinin (WGA) can be used as a probe to rapidly label migrasomes (154). The signal generated by WGA on migrasomes is much higher than that of RFs, and long-term exposure to WGA has little effect on the biosynthesis of migrasomes and cell migration, making it suitable for the rapid detection of migrasomes in cells (154). Further studies have found that RMG3, a small molecular fluorescent probe that binds to the lipid components of vesicle membranes, exhibits superior rapid and effective performance in migrasomes staining compared to WGA, and does not affect imaging quality after washing, which can be used to monitor the formation process of migrasomes in real time (155). Two-photon synthetic aperture microscopy (2pSAM) is a novel microscopic imaging technique that enables rapid three-dimensional imaging in deep tissues while reducing phototoxicity to live cells due to prolonged observation (41). For the first time, 2pSAM has rapidly and completely recorded the formation of germinal centers in lymph nodes during the immune response in mice with acute brain injury, as well as the generation of migrasomes and their mediated intercellular communication processes (41). Visualization imaging is of significant scientific value for understanding the morphological structure of migrasomes in pathophysiological processes.

#### 5.2.2 Biochemical characterization and analysis

Mass spectrometry can broadly screen protein markers associated with migrasomes, helping researchers understand their composition and function. Studies have shown that membrane proteins and cytoskeletal proteins are enriched in migrasomes when analyzed by mass spectrometry (156). These proteins are closely related to the processes of cell migration, cell adhesion, lipid metabolism, protein glycosylation, and glycoprotein metabolism, with expression levels in migrasomes being 1.5 times higher than in the cell body (156). Further analysis of migrasome-specific proteins revealed that, while TSPAN4 and integrin α5β1 are enriched in migrasomes and are critical for the formation, they are also present in exosomes and cannot serve as specific markers for identifying migrasomes (22). However, proteins such as NDST1, PIGK, CPQ, and EOGT are nearly undetectable in exosomes and can serve as specific markers for migrasomes, as well as key indicators to distinguish migrasomes from other vesicles (156). This finding provides new insights into the functions of migrasomes. In addition to specific proteins, migrasomes also contain cytokines and chemokines that play important roles in cell migration and the functions of surrounding cells. Techniques such as Western blotting (57), immunofluorescence (57) or immunohistochemistry (44) can be used to observe the co-localization of migrasomes with cytokines, indicating the expression of these cytokines within the migrasomes. Further detection of purified migrasome specimens using ELISA can provide quantitative analysis of cytokine expression levels (44). The functions of chemokines secreted by migrasomes can be evaluated through Transwell assays, scratch assays, and Boyden chamber assays (44). Additionally, PCR and quantitative lipidomics methods can be used to study mRNA expression (3) and lipid components (54) within migrasomes, aiding in a comprehensive understanding of their biological characteristics. In summary, biochemical characterization and analysis provide important tools and methods for studying the functions of migrasomes, which helps to reveal the potential role of migrasomes in diseases and their application prospects as biomarkers.

#### 5.2.3 Counting

By fluorescently labeling TSPAN4 or using WGA-coated magnetic beads in combination with flow cytometry, migrasomes can be precisely captured and effectively quantified (145). This method has been verified to be suitable for the count of migrasomes in plasma and urine, providing a reliable quantitative detection tool for clinical and basic research (123, 145). Nanoparticle tracking analysis is one of the commonly used methods in EVs research (114, 157, 158). NanoSight instrument can be used to accurately measure the size, distribution and concentration of migrasomes, so as to monitor the dynamic changes of migrasomes released by podocytes in urine (114, 144). Additionally, ImageJ software provides a tool for quantitative analysis of immunofluorescence results. By accurately assessing the fluorescence intensity of WGA, the expression levels of migrasomes can be reflected (33). These techniques establish a methodological foundation for the quantitative study of migrasomes, enabling researchers to compare the expression and function of migrasomes across different samples.

### 5.3 Challenges and limitations in clinical transformation of migrasomes

Despite the promising clinical applications of migrasomes, there are still certain issues regarding their clinical transformation. Currently, the isolation of migrasomes primarily relies on ultracentrifugation and density gradient centrifugation (2, 146–148). However, the abundance of migrasomes in complex body fluids such as blood is low, and they are easily co-precipitated with cell debris, resulting in insufficient purity. Traditional isolation methods have certain limitations when applied to clinical blood samples. Existing markers, such as TSPAN4 and PIGK, can differentiate migrasomes from exosomes, but their expression levels vary significantly in clinical samples and lack a unified threshold standard. Additionally, *in vivo* imaging techniques, such as 2pSAM can track the dynamics of migrasomes in real-time (41), but the high cost of equipment and the need for specialized operation limit their clinical application. Methods for the extraction, characterization, and quantification of migrasomes have yet to reach a consensus. Various migrasomes counting methods exhibit differences in sensitivity, leading to poor comparability of data between different studies. Furthermore, the preprocessing workflows for different clinical samples, such as blood and urine, lack standardization, which may introduce human error. While urinary migrasomes detection shows a promising biomarker for the early diagnosis of kidney disease with podocyte injury (144, 145), further multi-center large-sample validation is needed for it to serve as an independent diagnostic tool. Additionally, the biosafety of migrasomes, such as their immunogenicity and *in vivo* metabolic pathways, requires systematic evaluation.

In summary, visualization imaging techniques reveal the morphological structure of migrasomes, biochemical characterization analysis delves into migrasomes’ composition and function, while quantitative detection technologies provide reliable data support for clinical and basic research. By integrating these methods and techniques, we can conduct a comprehensive analysis of migrasomes, exploring their functions and dynamic changes within biological systems, and understanding their potential applications in immune regulation and diseases. This can ultimately provide important biomarker for the diagnosis and treatment of diseases. With the advancement of technology and the continuous development of new methods, we are expected to further improve the sensitivity and specificity of migrasomes detection methods, thereby better serving biomedical research and clinical practice.

## 6 Discussion and outlook

In recent years, migrasomes, as a unique type of extracellular vesicle, have opened up a new perspective. Although significant progress has been made in migrasomes research over the past decade, and their characteristics and functions have gradually been elucidated, the mechanisms of migrasomes formation remain incomplete, and how the vesicles are formed and transported into the migrasomes is still unclear. Furthermore, research on pathophysiology is also in the initial stage, and their roles in the onset and progression of clinical diseases remain to be further explored. There is also limited research on the smaller vesicles contained within migrasomes, and it is worth further exploring the roles these vesicles play within migrasomes. In summary, migrasomes hold promise as biomarkers for disease diagnosis and as delivery vehicles for therapeutic drugs in clinical applications. It is necessary to further understand the biological functions of migrasomes, particularly their roles in immune regulation. Exploring the roles of migrasomes in diseases and their potential as diagnostic biomarkers is an intriguing avenue for future research.

In this review, we summarize the roles of migrasomes in immune regulation and elucidate their biological functions in the interactions of immune cells during disease onset and progression (**Figure 2**). Overall, the research on migrasomes provides a new perspective on intercellular immune regulation. Migrasomes not only mediate early immune responses by activating immune cells (33), but also help maintain vascular homeostasis and promote tissue regeneration and repair (42). However, excessive immune activation may lead to damage in CAA (123). Additionally, migrasomes sustain the activity and function of immune cells through mitocytosis, thereby mediating immune homeostasis (32). Finally, migrasomes can also exert immunosuppressive effects. In the TME, migrasomes produced by tumor cells can promote the formation of an immunosuppressive microenvironment, aiding tumor cells in evading immune surveillance (133). By combining visualization imaging, biochemical characterization, and quantitative detection techniques, researchers can comprehensively reveal the structure, composition, and function of migrasomes, providing methodological support for further investigating their dynamic changes *in vivo* and their roles in immune regulation.

In summary, in-depth research on the biological functions of migrasomes in immune regulation will help uncover their mechanisms in the onset and progression of diseases, providing a theoretical basis for the development of new diagnostic and therapeutic methods. Furthermore, understanding the composition and function of migrasomes will offer new insights for personalized medicine, particularly in the treatment of tumors and immune-related diseases.
