# Supplementary material for: Migrasomes: key players in immune regulation and promising medical applications
Source: Front Immunol. 2025 May 15;16:1592314. doi: 10.3389/fimmu.2025.1592314 (PMC12119291; doi:10.3389/fimmu.2025.1592314)
Supplement: Supplementary file 1 [file Table1.docx]

Supplementary Material

**Supplementary Table 1. Distribution, source and contents of migrasomes in different pathophysiological states.**

| **Species** | **Pathophysiological states** | **Stimulant/inducer** | **Cell types/ tissues** | **Contents** | **Interacting/adhering cells/tissues/organs** | **References** |
| --- | --- | --- | --- | --- | --- | --- |
| zebrafish | zebrafish early embryonic development, organ morphogenesis and gastrulation | NA | embryonic cells | signalling molecules, including chemokines, morphogens, growth factors and cytokines, like integrin β1 and CXCL12 | embryonic shield cavity | (10) |
| chick | embryos angiogenes | NA | monocytes | TGF-β3, CXCL12 and VEGFA | chorioallantoic membrane of chicken embryos | (11) |
| human | bone marrow microenvironment | NA | mesenchymal stromal cells | leukocyte cell adhesion molecule (ALCAM, CD166), SDF-1, CXCL12, Rab7 and CD63 | KG-1a leukemic cells and primary CD34+ hematopoietic progenitors | (12) |
| human | hypoosmotic stress | hypoosmotic stress | H4 human neuroglioma cells | cytoplasmic components | NA | (13) |
| mouse, human | miscarriage | PS-NPs, 50 nm (suppress) | trophoblast cells | NA | NA | (14) |
| mouse | acute ischemic stroke | high-salt diet | microglia/macrophages | neuronal fragments | surrounding neurons and white matter | (15) |
| mouse | post‐stroke pneumonia | bacterial stimulation | bone marrow mesenchymal stem cells | antibacterial peptide dermcidin | macrophages | (16) |
| mouse, human | podocyte injuries, PAN-nephropathy and diabetic nephropathy | LPS, puromycin amino nucleoside, or a high concentration of glucose | podocytes | NA | kidney | (17) |
| mouse, human and rabbit | proliferative vitreoretinopathy | TGF-β1/Smad2/3 signaling pathway | retinal pigmented epithelium | NA | retinal pigmented epithelium | (18) |
| mouse | microbial infections | Clostridioides difficile toxins TcdB3 | liver sinusoidal endothelial cells and kupffer cells | cytokines and chemokines, including CXCL10, CCL5, VEGFA, and IL-10 | hepatic blood vessels and sinusoids | (19) |
| mouse | antigen uptake | FAAs | DCs | FAAs | NA | (20) |
| mouse | localized inflammation | LPS | monocytes | TNF-α and IL-6 | accumulation at the site of inflammation | (21) |
| human | virus infection | CHIKV-nsP1 | Hela cells(transfected with plasmid CHIKV-nsP1 | CHIKV-nsP1 | NA | (22) |
| mouse, human | wound | wound | neutrophil | coagulation factors, including prothrombin, factor XIII B, factor X, factor VIII, factor XI, factor XII and Von Willebrand factor，and cholesterol ester | activate platelets and accumulate at injury sites | (23) |
| mouse, human | CAA | Aβ40 | macrophage lineage cells | CD5L | brain blood vascular and endothelial cells | (24) |
| mouse | traumatic brain injury or closed head injury | acute brain injury | neutrophil | NA | microglia | (25) |
| human | glioblastoma | ER stress | glioblastoma cells | LC3B-positive autophagosomes, ER-associated proteins | NA | (26) |
| mouse, human | pancreatic cancer | NA | pancreatic cancer cells and tissues | chemokines, cytokines and signaling molecules, including CX3CL1, CXCL5, CCL1, CCL27, CCL21, TGF-β1, TGF-β2, ITGA2, ITGA3, ITGB1, ITGB7, Rab2b and Rab 32 | induced an immunosuppressive phenotype of macrophages and promoted pancreatic cancer progression | (27) |
| human | hepatocellular carcinoma (HCC) | NA | HCC cell lines: HCCLM3 and MHCC97H | CD151 and VEGF | neighbouring liver cancer cells | (28) |
| human | tumor bone metastasis | tumor bone metastasis | tumour cells（4T1 cells） | RNA | mediate the tumour–TAOC ‘tumasteoclast’ coupling | (29) |

Abbreviations: Aβ40, amyloid protein beta 1-40; ALCAM, activated lymphocyte cell adhesion molecule; CAA, cerebral amyloid angiopathy; CCL5, c-c motif chemokine ligand 5; CD166, cluster of differentiation 166; CD5L, CD5 antigen-like; CD63, cluster of differentiation 63; CHIKV, Chikungunya virus; CXCL12, chemokine (C-X-C motif) ligand 12; CX3CL1, c-x3-c motif chemokine ligand 1; DCs, dendritic cells; ER, endoplasmic reticulum; FAAs, fluorescent artificial antigens; HCC, hepatocellular carcinoma; IL-10, interleukin 10; ITGA2/B1, integrin alpha-2/beta-1; LC3B, microtubule-associated protein 1a/1b; LPS, lipopolysaccharide; NA, not applicable; PAN, puromycin amino nucleoside; PS-NPs, polystyrene nanoplastics; SDF-1, stromal cell-derived factor 1; TAOC, tumour-associated osteoclast; TcdB3, clostridium difficile toxin b variant 3; TGF-β, transforming growth factor beta; VEGF, vascular endothelial growth factor.
